# Headaches in Children

**Published:** 1882-06

**Authors:** 


### HEADACHES IN CHILDREN.

Dr. Day read a paper on this subject before a recent meeting of the Harveian Society, of London (London *Lancet*). He alluded to the two great causes of headache, from a pathological point of view—viz. cerebral anæmia and cerebral hyperæmia. He said that habitual headaches in children indicate an irritable and exhausted brain, and if intellectual exertion be carried too far in such cases, mischief is soon likely to ensue. If intellectual exertion be carried beyond a certain point, the brain becomes anæmic, fatigued, and the nutrition in the ganglion cells of the cortex becomes impaired, diseased, or in some way altered from health. The author referred to neuralgia, or one-sided headache, which he said was more common in children than was generally supposed. He had known headache in connection with chorea and dental caries. Dr. Cheadle considered foul air and gas were the chief causes of study-headache; he referred to headaches in rickety children. This occurred just after the skull closed, was continuous, but gradually subsided. Dr. Mackenzie particularly insisted on the importance of careful examination of the eyes in cases of headache of children. Muscular asthenopia was a cause of headache which sometimes was mistaken for serious organic disease. He mentioned the case of a schoolboy brought to him under this supposition, but myopia was found and corrected, and the headache disappeared. The same thing occasionally occurred with hypermetropia. Next he pointed out that ear disease was sometimes the cause of headache; which is important as significant of commencing meningeal or cerebral inflammation. In all cases, therefore, of headache in children, it is very important to examine the ear and the eye, using the ophthalmoscope, which will be of great value in detecting organic disease. He remarked that pain in the head, a valuable sign of tumor of the brain, was no certain indication of localization of the tumor, unless there was corresponding pain on percussion. The President, in conclusion, said that headache, though not a common symptom in children, was one of import, and frequently indicated advanced disease.

